# Synergistic Antibacterial and Pro-Healing Effects of a Novel Eugenol/Nano-*Haliotidis Concha* Electrospun Membrane for *Vibrio vulnificus*-Infected Wound

**DOI:** 10.3390/polym18060704

**Published:** 2026-03-13

**Authors:** Fuyu Zhao, Xianjun Fu, Wuyi Zhou, Xia Ren

**Affiliations:** 1 Research Institute for Marine Traditional Chinese Medicine (Qingdao Academy of Chinese Medical Sciences), The SATCM’s Key Unit of Discovering and Developing New Marine TCM Drugs, Key Laboratory of Marine Traditional Chinese Medicine in Shandong Universities, Shandong University of Traditional Chinese Medicine, Jinan 250355, China; 2023111578@sdutcm.edu.cn (F.Z.); fuxianjun@sdutcm.edu.cn (X.F.); 2 Qingdao Academy of Chinese Medical Sciences, Shandong University of Traditional Chinese Medicine, Qingdao 266114, China; 3 College of Materials and Chemical Engineering, Guangdong Province Biobased Green Packaging Materials Engineering Technology Research Center, South China Agricultural University, Guangzhou 510642, China

**Keywords:** *Haliotidis Concha* (HC), fibrous membrane, *Vibrio vulnificus*, wound healing

## Abstract

Wounds caused by *Vibrio vulnificus* (*V. vulnificus*) infection often exhibit delayed healing and are prone to complications, making them a significant challenge in clinical treatment. Current conventional treatments, such as antibiotics and gauze dressings, have limited effectiveness. To address this, this study developed a multifunctional fiber membrane using electrospinning technology. Micron- or nano-sized *Haliotidis Concha* (HC) and eugenol (Eu) were loaded onto the membrane to promote healing in *V. vulnificus*-infected wounds. The prepared fiber membranes exhibited diameters of approximately 0.35 ± 0.01 μm. Membranes loaded with nano-HC demonstrated significant antibacterial efficacy, achieving a 96.2% inhibition rate against *V. vulnificus*, which was markedly superior to the micron-HC group (*p* < 0.05). Notably, the nano-HC/Eu membranes exhibited exceptionally high flexibility with an elongation at break of 878.1 ± 35.3%, while maintaining a tensile strength of approximately 2.2 MPa. Furthermore, these membranes exhibited excellent biocompatibility, with cell viability exceeding 85% for fibroblasts, and demonstrated good hemocompatibility. They also effectively promoted cell migration, indicating their potential as wound scaffold materials. In a *V. vulnificus*-infected skin wound model, the nano-HC/Eu fiber membrane accelerated collagen deposition and promoted wound healing, achieving a wound closure rate of 94.7 ± 1.1% on day 15. In summary, this study developed a multifunctional fiber membrane with antibacterial, antioxidant, and wound healing properties, offering a novel dressing for treating *V. vulnificus* infections.

## 1. Introduction

Traumatic injuries sustained by offshore workers in marine environments often heal more slowly than those occurring on land, primarily due to the heightened risk of bacterial infection in the marine environment [1]. Infection by marine bacteria aggravates edema and promotes tissue degeneration and necrosis in the peri wound region. These pathological changes impede wound repair and contribute substantially to healthcare costs worldwide [2]. Among marine pathogens, *Vibrio vulnificus* (*V. vulnificus*) is a primary threat responsible for severe wound infections in coastal areas [3]. *V. vulnificus* infections progress rapidly, potentially causing severe gastrointestinal disease, sepsis, and even life-threatening conditions. These infections present a serious challenge for clinical prevention and treatment [4]. As global warming intensifies, the number of patients suffering from *V. vulnificus* infections continues to rise daily [5,6], with increasingly severe symptoms, posing a significant risk to human health [7]. The current clinical management of *V. vulnificus* infections primarily relies on prompt surgical debridement and aggressive antibiotic therapy [8]. However, the emergence of antibiotic-resistant *V. vulnificus* strains has been increasingly reported, compromising treatment efficacy [9,10]. Conventional wound dressings, such as gauze, primarily serve as physical barriers and lack inherent antimicrobial activity, failing to prevent bacterial colonization or promote tissue regeneration [11]. Although silver-containing dressings have been widely used due to their broad-spectrum antimicrobial properties, their potential cytotoxicity limits their long-term application in wound healing [12]. Bacterial infection is a critical factor that severely impedes the wound healing process [13]. Addressing *V. vulnificus* infections requires the development of a safe and effective therapeutic approach to promote wound healing and improve patient outcomes.

Eugenol (Eu) is a natural compound extracted from plants belonging to the orchid, laurel, myrtle, and nutmeg families, possessing broad-spectrum potent antibacterial activity [14]. Studies indicate that Eu exhibits excellent inhibitory effects against *V. vulnificus* [15]. Eu exhibits antibacterial activity against *V. vulnificus* through multiple mechanisms, including inducing oxidative stress, altering membrane potential, disrupting cell membrane integrity, modifying cell morphology, and eliminating biofilms [15]. Although Eu exhibits broad-spectrum and potent antibacterial activity, it lacks biological functions that promote tissue repair and regeneration. This limitation may hinder the synergistic promotion of antibacterial action and the healing process in the clinical management of infected wounds, thereby affecting the ultimate efficacy of restoring tissue integrity. Marine traditional Chinese medicine (MTCM) refers to drugs derived from marine sources for the prevention, diagnosis and treatment of diseases. *Haliotidis Concha* (HC), also known as Shijueming, is the shell of the abalone mollusk and represents a typical MTCM. Its primary component is calcium carbonate, supplemented by trace elements such as calcium, magnesium, iron, and zinc, along with amino acids. Modern pharmacology studies reveal that HC also exhibits hypotensive [16], antibacterial, antioxidant [17], and gastric acid neutralizing effects, and also promotes wound healing [18]. Calcium ions play an essential role in the hemostatic stage of wound repair by acting as a key cofactor in the blood coagulation cascade. It not only assists platelet aggregation to form platelet plugs but also activates the coagulation pathway [19]. However, the therapeutic efficacy of unmodified HC is often limited by its low bioavailability and insufficient interaction with wound tissues [20]. Nanoparticle processing can substantially increase its specific surface area, enhancing interactions with bacteria and wound tissues. Grinding HC into nanoparticles effectively increases their specific surface area and biological activity [21], offering a potential solution to this challenge.

However, most current dressings fail to simultaneously address rapid bacterial proliferation, oxidative stress, and impaired tissue regeneration under marine infection conditions. In marine environments, open skin injuries are continuously exposed to high humidity and elevated salinity. They are also vulnerable to pathogenic microorganisms such as *V. vulnificus*, a bacterium characterized by rapid proliferation, strong invasiveness, and pronounced tissue destructive capability. Under such complex conditions, traditional dressings and antimicrobial materials designed primarily for terrestrial pathogens often struggle to meet clinical needs. These materials usually only provide basic physical isolation and have limited effects in terms of inhibiting bacterial colonization [22]. They also lack the ability to modulate the inflammatory response in the early phase of wound healing. Moreover, the absence of multifunctional synergistic mechanisms, such as antimicrobial, antioxidant, and healing-promoting activities, makes it difficult to effectively manage complex infected wounds. In recent years, new drug delivery platforms such as fibrous membranes, hydrogels, and microspheres have received widespread attention and applications in the biomedical field. These platforms offer excellent drug loading efficiency, good biocompatibility, controlled release properties and outstanding physicochemical stability [23]. Ensuring biocompatibility is essential for antimicrobial materials intended for biomedical applications. Fibrous membranes are particularly promising as wound dressings due to their unique structural features. They possess an extremely high specific surface area and an interconnected porous structure [24]. These characteristics enable efficient drug loading and sustained release. Moreover, the fibrous architecture closely mimics the topological features of the native extracellular matrix. This provides a favorable microenvironmental scaffold that supports cell adhesion, migration, proliferation, and differentiation at the wound site [23,25]. By loading antibiotics, natural antimicrobial ingredients or growth factors onto the fibrous membranes, smart wound dressings can be constructed. These dressings combine long-lasting antimicrobial [26], proangiogenic and tissue regenerative functions. As a result, they effectively control infection, promote tissue repair, and accelerate the healing of complex wounds.

Therefore, this study aims to develop a multifunctional electrospun fibrous membrane for treating *V. vulnificus*-infected wounds. The membrane is based on Polycaprolactone/Polyethylene glycol (PCL/PEG) and incorporates eugenol (Eu) along with micron- or nano-sized HC. This combination is designed to synergistically regulate the infected wound microenvironment and promote wound healing. PCL is a biodegradable polyester with excellent mechanical properties and biocompatibility, making it one of the most widely used polymers for electrospun wound dressings [27]. Its relatively slow degradation rate provides long-term structural support during the wound healing process. PEG, a hydrophilic and nontoxic polymer, was incorporated to modulate the hydrophilicity and flexibility of the fibers, as well as to improve the biocompatibility of the membrane [28]. The marine medicinal substance HC promotes wound healing, while Eu effectively inhibits *V. vulnificus* in marine environments. This work aims to develop advanced multifunctional wound dressings for infections caused by marine pathogens. It also provides experimental evidence for the application of marine traditional Chinese medicines in tissue reconstruction.

## 2. Materials and Methods

### 2.1. Materials

HC was procured from Xuyi Pharmaceutical Co., Ltd. (Huaian, China); PCL (Mw = 50,000 g/mol) and PEG (Mw = 2000 g/mol) were purchased from McLean Biotechnology Co., Ltd. (Singapore); Eu (≥99% purity) was acquired from Aladdin Reagent Co., Ltd. (Shanghai, China); dichloromethane (DCM, ≥99.9% purity) and dimethylformamide (DMF, ≥99.5% purity) were purchased from Tianjin Fuyu Fine Chemical Co., Ltd. (Tianjin, China); and phosphate-buffered saline (PBS, pH = 7.4) was provided by White Shark Biotechnology Co., Ltd. (Singapore).

### 2.2. Preparation of Micron- and Nanoscale HC

The micron-sized HC was prepared as follows. HC was pulverized into a coarse powder in a high-speed multipurpose grinder. The sieved powder was mixed with deionized water at a 1:15 solid-to-liquid ratio, and then ground for 1 h at 500 rpm in a planetary ball mill (F-P4000, Hunan Fucas Laboratory Instrument Co., Ltd., Changsha, China). After grinding, the mixture was collected and freeze-dried to obtain micron-sized HC powder.

The nano-sized HC was prepared as follows. The coarse powder was ultrasonically ground (KDP0.5, Guangdong Pailer Intelligent Nano Technology Co., Ltd., Guangzhou, China) at a solid-to-liquid ratio of 1:20 and a rotation speed of 3000 rpm for 5 h. During grinding, sodium hexametaphosphate was introduced as a dispersant. After grinding, the mixture was collected and freeze-dried to produce nano-sized HC. The preparation method for micron- and nano-sized HC was adapted from a previously reported protocol by Huang et al. [20].

### 2.3. Preparation of Fibrous Membranes

First, 2.8 g of PCL was dissolved in 20 mL of DCM/DMF (volume ratio of 3:1) to obtain the PCL transparent spinning solution. PCL and PEG were combined at a weight ratio of 8:2 in the same DCM/DMF (volume ratio of 3:1) solvent. The mixture was stirred magnetically for 24 h at room temperature to obtain the PCL/PEG spinning solution. Then, 0.2 g of micron-sized or nano-sized HC and 100 µL of Eu were added to the PCL/PEG solution. After continuous stirring for 2 h at 25 °C, the mixture was ultrasonicated for 30 min. Finally, electrospinning was performed using a far field electrospinning apparatus (TL-01, Shenzhen Tongli Micro-Nano Technology Co., Ltd., Shenzhen, China). The spinning solution was loaded into a disposable sterile syringe, air bubbles were expelled, and an 18G stainless steel flat tip needle was attached. The electrospinning conditions were as follows: an applied voltage of 20 kV, a solution feed rate of 2 mL/h, a needle to collector distance of 18 cm, and an ambient environment maintained at 25 °C with 40% relative humidity. This process produced the fibrous membranes.

### 2.4. Morphological Characterization of Fibrous Membranes

The morphology of the fibrous membranes was examined with scanning electron microscopy (SEM, EVO MA 15, ZEISS, Eschborn, Germany). The membranes were cut into appropriate sizes, mounted onto an SEM stage, sputter-coated with gold, and then their surface morphology was examined by SEM. The diameters of 30 randomly selected fibers were measured using ImageJ software (version 1.54p, National Institutes of Health, Bethesda, ML, USA), and the results were expressed as the mean ± standard deviation.

The morphology of HC in the fiber membrane was observed using a transmission electron microscope (TEM, Thermo Fisher Talos F200S G2, Waltham, MA, USA). First, the fiber membranes were ultrasonically dispersed and applied onto a grid, after which they were allowed to air dry naturally. Subsequently, the grids were mounted onto a sample holder and inserted into the instrument. During imaging, the fiber dispersion areas were first located in LowMAG mode, and the morphology was captured by switching to MAG mode.

### 2.5. Fourier Transform Infrared (FTIR) Spectroscopy of Fibrous Membranes

The chemical structure of the fibrous membranes was characterized by FTIR spectroscopy (Nicolet IS10, Thermo Fisher Scientific, Waltham, MA, USA) over the range of 400–4000 cm^−1^, using a spectral resolution of 4 cm^−1^ and 32 scans per sample.

### 2.6. Mechanical Properties of Fibrous Membranes

The mechanical properties of fibrous membranes were evaluated using a Shimadzu AGS-X universal testing machine (Shimadzu Corporation, Tokyo, Japan). The membrane samples (10 mm × 90 mm rectangular strips) were stretched at 10 mm/min. For each type of membrane, five independent samples (*n* = 5) were tested, and the results were expressed as the mean ± standard deviation.

### 2.7. Thermal Stability Testing of Fibrous Membranes

Thermal stability testing was conducted using a thermogravimetric analyzer (TGA, TG209F1 Libra TM, Nerz Instruments Manufacturing GmbH, Selb, Germany) and differential thermogravimetric analysis (DTGA). A precise mass of the fiber sample was weighed using an analytical balance and placed in a crucible. Under a nitrogen atmosphere, the samples were heated from 35 to 900 °C at a rate of 10 K/min, and their mass loss curve was recorded.

### 2.8. Water Vapor Barrier and Surface Hydrophilicity

#### 2.8.1. Water Contact Angle Testing of Fibrous Membranes

A water contact angle goniometer (OCA20, Shanghai Zhongchen Digital Technology Equipment Co., Ltd., Shanghai, China) was used to evaluate the hydrophilicity of the membranes. A water droplet was placed on the membrane surface using a syringe, and the static contact angle at the solid–liquid interface was recorded.

#### 2.8.2. Water Vapor Transmission Testing of Fibrous Membranes

The water vapor transmission rate (WVTR) of the fibrous membranes was determined following the method described by Yin et al. [29] with minor modifications. Briefly, 10 g of deionized water was added to a 30 mL sample vial. The vial opening was covered with a membrane of similar thickness and secured tightly with a rubber band. Two additional sets of sample bottles were prepared. One set was covered with PE plastic wrap and served as the control group, while the other set was left uncovered as the blank control. The weight of the water in the sample bottles was recorded after 24 h at room temperature. Three parallel samples (*n* = 3) were tested for each membrane type, and the average WVTR value was calculated. The WVTR of the fibrous membranes was determined according to the following equation, with the unit being g·m^−2^·d^−1^:

(1) $$\mathrm{WVTR}=\frac{W_{1}-W_{0}}{S}\times 100\%$$

W_0_ is the initial weight of water in the sample bottle (g); W_1_ is the weight of water after 24 h (g); and S is the opening area (m^2^).

### 2.9. Antimicrobial Testing of Fibrous Membranes

The antibacterial activity of various fibrous membranes against *V. vulnificus* was evaluated using the plate count method. The activated *V. vulnificus* was diluted in sterile saline to 1 × 10^7^ CFU/mL. The membranes were sterilized by UV irradiation and then added to the diluted *V. vulnificus* suspension. The container was sealed and incubated for 6 h at 30 °C in a shaker (110 rpm/min). After incubation, 0.1 mL of the bacterial suspension was pipetted onto the surface of solid culture medium. The plates were inverted and incubated at 30 °C for 20 h. Colony growth on the surface of the medium was observed and the data were recorded. All experiments were performed with three independent samples (*n* = 3) for each group to ensure reproducibility. The antibacterial rate for each fiber membrane group was calculated as follows:

(2) $$\mathrm{Antibacterial}\;\mathrm{rate}=\frac{N_{1}-N_{2}}{N_{1}}\times 100\%$$

N_1_ represents the colony count in the blank control group; N_2_ represents the colony count for fibrous membrane samples.

### 2.10. Antioxidant Testing of Fibrous Membranes

The antioxidant capacity of the fibrous membrane was evaluated using the DPPH radical scavenging assay. The procedure was performed as follows. Fiber membrane samples were cut into 1 cm × 1 cm squares and were placed in sample vials containing 5 mL of DPPH solution and allowed to react for 30 min at room temperature under dark conditions. The absorbance of the test solution was measured at 517 nm using a UV-Vis spectrophotometer (Mettler Toledo, Singapore). Finally, the DPPH radical scavenging activity was determined according to the following equation using the measured absorbance values:

(3) $$\mathrm{DPPH}\;\mathrm{scavening}\;\mathrm{rate}=\frac{A_{D}-A_{F}}{A_{D}}\times 100\%$$

A_D_ is the absorbance of the DPPH solution (a.u.); A_F_ is the absorbance of the fiber membranes’ groups (a.u.).

### 2.11. Eu Release Testing of Fibrous Membranes

Eu release studies were conducted to quantify the amount of Eu released from the fiber membranes. Briefly, membrane samples were placed in test tubes with a surface area-to-volume ratio of 3 cm^2^/mL. They were then immersed in PBS containing 0.2% (*v*/*v*) Tween 80 to promote Eu release. The samples were incubated at 37 °C. At predetermined time points, the supernatant was collected and replaced with an equal volume of fresh PBS containing 0.2% (*v*/*v*) Tween 80. The Eu concentration in each collected aliquot was determined by UV-Vis spectrophotometry at 282 nm. The cumulative release percentage was calculated as follows:

(4) $$\mathrm{Cumulative}\;\mathrm{release}\;\mathrm{rate}=\frac{C_{n}V+\sum_{i=1}^{n}C_{n-1}V}{M_{0}}\times 100\%$$

C_n_ denotes the Eu concentration at the n-th sampling time; V is the total volume of the release medium; v is the aliquot volume withdrawn at each sampling time; and M_0_ is the initial Eu content in the fiber membranes.

### 2.12. Cytotoxicity Assay

Cellular activity was evaluated using L929 mouse fibroblasts (cells were kindly provided by Suzhou Haixing Biosciences Co., Ltd., Suzhou, China). L929 cells were seeded at a density of 5 × 10^3^ cells per well in a 96-well plate. The fibrous membranes were sterilized by UV irradiation and then immersed in complete medium at an area-to-volume ratio of 3 cm^2^/mL. The samples were incubated for 24 h to promote drug release. The membranes extracts were co-cultured with L929 cells for 24 h and 48 h. Cells’ viability was evaluated using a Cell Counting Kit-8 (CCK-8) assay (Dojindo Molecular Technologies, Rockville, ML, USA). After incubation, 10 μL of CCK-8 reagent was added to each well and continue incubation for 2 h. Absorbance was measured using a microplate marker at 450 nm at the end of incubation. All the experiments were performed with three independent samples (*n* = 3) for each group to ensure reproducibility. The calculation of cell viability was performed as follows:

(5) $$\mathrm{Cell}\;\mathrm{viability}=\frac{{\mathrm{OD}}_{s}-{\mathrm{OD}}_{b}}{{\mathrm{OD}}_{c}-{\mathrm{OD}}_{b}}\times 100\%$$

OD_s_ is the absorbance of the experimental group; OD_b_ is the absorbance of the negative control group; and OD_c_ is the absorbance of the positive control group.

### 2.13. Cell Scratching Assay

The capacity of the fibrous membranes to promote cell migration was evaluated using a scratch assay with L929 mouse fibroblasts. The cells were seeded into 6-well culture plates. After adhesion, linear scratches were created using a sterile 200 μL pipette tip. Detached cells were removed by gently rinsing the wells three times with PBS. Cell migration was monitored and imaged at 0, 6, and 12 h. All the experiments were performed with three independent samples (*n* = 3) for each group to ensure reproducibility. The migration area was quantified using ImageJ software.

(6) $$\mathrm{Cell}\;\mathrm{migration}\;\mathrm{rate}=\frac{C_{0}-C_{t}}{C_{0}}\times 100\%$$

C_0_ is the cell scratch area at time 0 h; C_t_ is the cell scratch area at time t h.

### 2.14. Hemocompatibility of Fibrous Membranes

The hemolysis test was used to assess the hemocompatibility of the fibrous membrane. Sodium heparin-anticoagulated murine whole blood was centrifuged at 3000 rpm for 10 min. The supernatant was gently aspirated, and the erythrocytes were washed by adding PBS. The collected erythrocytes were prepared as a 5% (*v*/*v*) suspension for subsequent use. The fiber membrane was immersed in PBS at a ratio of 3 cm^2^/mL for 24 h to release the drug. A 0.5 mL aliquot of the diluted erythrocyte suspension was mixed with 0.5 mL of the test solution and incubated in a 37 °C water bath for 1 h. The mixture was then centrifuged at 3000 rpm for 10 min, and the supernatant was carefully collected. The absorbance of the supernatant was measured at 540 nm using a microplate reader. The hemolysis ratio of the membranes was calculated using the following equation:

(7) $$\mathrm{Hemolysis}\;\mathrm{rate}=\frac{{\mathrm{OD}}_{s}-{\mathrm{OD}}_{b}}{{\mathrm{OD}}_{c}-{\mathrm{OD}}_{b}}\times 100\%$$

OD_s_ is the absorbance of the experimental group; OD_b_ is the absorbance of the negative control group; and OD_c_ is the absorbance of the positive control group.

### 2.15. In Vivo Healing Experiment with Fibrous Membranes

The wound healing potential of the fiber membrane was evaluated using a mouse model of *V. vulnificus* infection. All animal procedures were conducted in accordance with the protocol approved by the Laboratory Animal Ethics Committee of Shandong University of Traditional Chinese Medicine (No. SDUTCM20251024101). To investigate the wound healing potential of the membranes, male BALB/c mice (8 weeks old, weight 20–25 g, and all purchased from Shandong Pengyue Laboratory Animal Technology Co., Ltd., Jinan, China) were acclimatized for 7 days prior to experimentation under the standard conditions of 25 °C, a 12 h light/dark cycle, and with free access to water and food. Twenty-four mice were randomly divided into four groups (*n* = 6 per group): a blank control group receiving no treatment; a positive control group treated with 3 M Tegaderm transparent dressing; a micron-HC/Eu fiber membrane group, and a nano-HC/Eu fiber membrane group. This sample size represents the minimum number required to obtain statistically reliable results. It also adheres to the principle of the ‘3R’ in animal use. After the dorsal area was shaved and the animals were anesthetized, a 10 mm full-thickness excisional wound was created on the dorsum. An activated *V. vulnificus* suspension was diluted with sterile saline to 10^8^ CFU/mL. A volume of 100 µL of the bacterial suspension was then applied to each wound surface. After allowing 15–30 min for complete bacterial adsorption, the wound was covered with 3M Tegaderm transparent dressing. After 24 h of infection, the wounds were treated with different dressings. Wounds were assessed and photographed on days 0, 3, 7, 11, and 15. The wounds’ healing rates were analyzed using ImageJ software. In addition, to quantitatively evaluate the in vivo antimicrobial properties of the fiber membranes, wound tissues from each group were collected after 3 days of treatment. The tissues were homogenized, serially diluted, and plated on agar for colony counting to determine the bacterial load. On day 15, wound tissues were collected, fixed in 4% paraformaldehyde, embedded in paraffin, and sectioned. The sections were subjected to histological analysis using hematoxylin and eosin (H&E) and Masson’s trichrome staining. This study followed the ARRIVE guidelines. Humane endpoints were predefined throughout the experiment in accordance with institutional guidelines (see Supplemental Information for detailed criteria). The wounds’ healing rates were calculated from the wound area using the following formula:

(8) $$\mathrm{Wound}\;\mathrm{healing}\;\mathrm{rate}=\frac{A_{0}-A_{t}}{A_{0}}\times 100\%$$

A_0_ denotes the initial wound area (cm^2^), whereas A_t_ denotes the wound area (cm^2^) at t points.

### 2.16. Statistical Analysis

SPSS 23.0 software was used to perform normality and homogeneity of variance tests on raw data. For data meeting the normality and homogeneity of variance criteria, one-way analysis of variance (ANOVA) and Tukey’s post hoc multiple comparisons were conducted. Origin 2022 was used for graphing. Image analysis was performed using ImageJ software. All the quantitative results are reported as the mean ± standard deviation. Statistical significance was assessed using the following thresholds: * *p* < 0.05, ** *p* < 0.01, and *** *p* < 0.001.

## 3. Results and Discussion

### 3.1. Structural and Physicochemical Characteristics of the Fibrous Membranes

**Figure 1:**
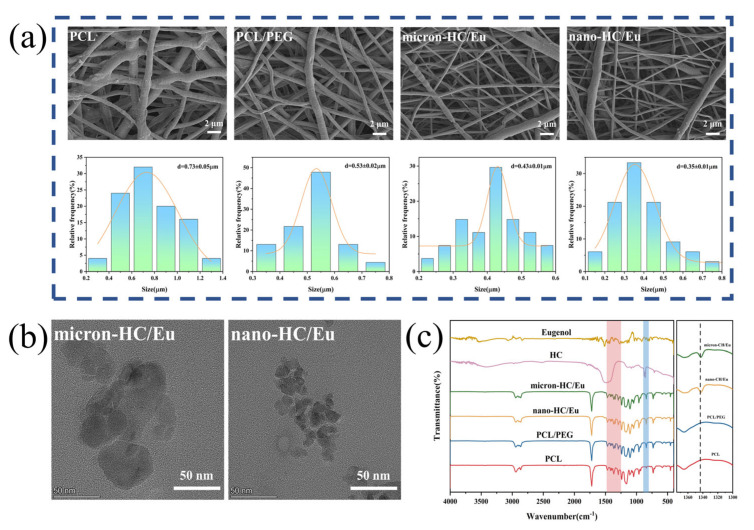
Fibrous membrane performance analysis: (**a**) microstructural morphology and fiber diameter analysis of fibrous membranes; (**b**) TEM images of the fiber membrane; and (**c**) FTIR spectra of membranes, HC and eugenol.

The fibrous membranes were fabricated using a high-voltage electrospinning device. The optimization process for the fibrous membrane formulation was provided in the Supporting Information (Table S2; Figures S3–S5). All membranes exhibited an interconnected fibrous network with good overall fibrous continuity and no obvious breakage or beading (Figure 1a). The average diameter of the PCL fibrous membranes was mainly concentrated in the range of 0.73 ± 0.05 μm, which decreased to 0.53 ± 0.02 μm after the incorporation of PEG. Notably, the nano-HC/Eu fibrous membranes had a much smaller average diameter (0.35 ± 0.01 μm) compared to micron-HC/Eu fibrous membranes (0.43 ± 0.01 μm). The high viscosity and poor electrical conductivity of the PCL solution resulted in insufficient stretching during spinning and ultimately cured thicker fibrous [30,31]. The addition of PEG, HC and Eu reduces the diameter of the membrane, which may be due to the reduced viscosity of the mixed solution and increased electrical conductivity (Figure S6) [32,33]. The synergistic effect of reduced viscosity and increased conductivity significantly promoted fiber draw-out and refinement. As reported previously, the smaller the fiber diameter, the more it promotes cell adhesion, proliferation, and migration, which in turn regulates the cell cycle and enhances cell proliferation [34].

TEM was employed to characterize the microstructures of HC within the fiber membranes with the results shown in Figure 1b. The TEM images clearly revealed the loading states of particles on the fiber membranes. HC particles on the nano-HC/Eu fiber membranes primarily exhibited a blocky morphology, with diameters distributed within the range of 9–16 nm and an average particle size of approximately 12.9 nm. This fine nanoscale endowed the fiber membranes with unique physicochemical properties. From the perspective of wound dressing applications, the small size effect of nanoparticles in nano-HC/Eu fiber membranes delivered significant performance advantages. Compared to micron-sized particles, nanoparticles exhibited a substantially increased specific surface area. This effectively enhanced the bioavailability of HC, enabling more efficient wound healing promotion and accelerating the wound repair process.

FTIR spectroscopy was employed to further confirm the chemical structure of the fiber membranes and verify the incorporation of HC and Eu. Figure 1c shows that the absorption peak at 840 cm^−1^ is the characteristic absorption peak of calcium carbonate, the main component of HC. Aromatic ring skeletal vibrations were observed at 1472 cm^−1^, 1419 cm^−1^, and 1341 cm^−1^, while an absorption band at 1269 cm^−1^ was assigned to the asymmetric C-O-C stretching vibration [35]. These bands were characteristic absorption peaks of Eu. These characteristic peaks were consistently observed in the spectra of the composite, confirming the successful incorporation of both HC and Eu into the membranes.

### 3.2. Mechanical Performance and Thermal Stability for Wound Dressing Applications

**Figure 2:**
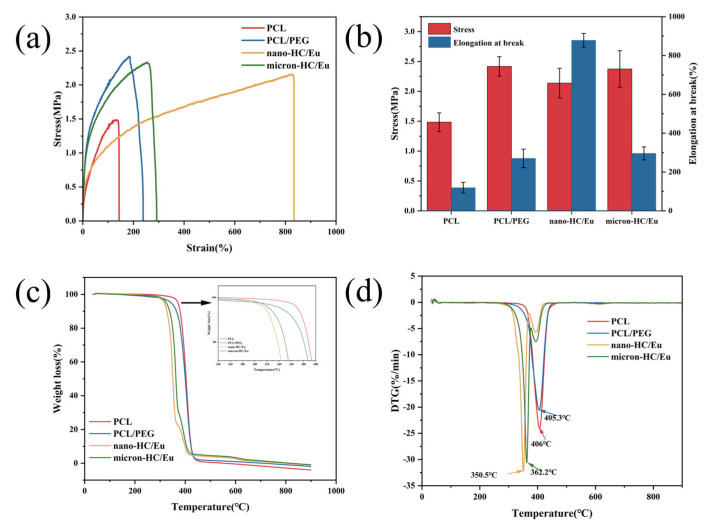
Mechanical characterization and thermal analysis of the fiber membrane: (**a**) stress–strain curves of fibrous membranes; (**b**) bar chart of stress and elongation at break for the membrane (**c**) TG of membranes; and (**d**) DTG of membranes.

Electrostatically spun fibrous membranes need to have good mechanical properties when used as wound dressings. The stress–strain curves and elongation at break of the various fiber membranes are shown in Figure 2a,b. The PCL membrane exhibited a tensile strength of 1.4 ± 0.2 MPa and an elongation at break of 118.9 ± 27.3%. The incorporation of both nano-HC/Eu and micron-HC/Eu significantly elevated tensile strength to about 2.1 ± 0.2 MPa and 2.4 ± 0.3 MPa, respectively, indicating enhanced mechanical reinforcement. Notably, the nano-HC/Eu membrane exhibited the highest elongation at break (878.1 ± 35.3%), demonstrating exceptional flexibility and elasticity. This might be due to the fact that fiber membranes containing nano-HC/Eu benefit from a smaller diameter and uniform distribution of fibrous [36]. The simultaneous enhancement of fiber membrane elongation and tensile strength might be attributed to the effective stress transfer facilitated by nano-HC particles as reinforcing phases [37]. The refinement of fiber diameter constructs a denser network, increasing load distribution points [38]. Concurrently, improved fiber uniformity reduces structural defects that act as stress concentration points [39]. Collectively, these factors enable the material to withstand higher stresses and strains. Such high flexibility is particularly advantageous for wound dressings applied to moving body parts or irregular wound surfaces, as it allows the material to conform to the wound bed without tearing or causing discomfort.

The thermal stability of the membranes was assessed by TGA and DTGA. Figure 2b,c show that the PCL membrane exhibited an initial degradation temperature (defined as 5% weight loss, T_5%_) of 358.4 °C, and reached the maximum decomposition temperature rate (T_max_) at 406 °C. After the addition of PEG, the T_5%_ of membranes did not change significantly, and the T_max_ was kept unchanged, which showed that PEG decomposed together with PCL. The T_5%_ of the nano-HC/Eu membrane was 318.3 °C, which was lower than that of PCL and PCL/PEG, and its T_max_ appeared at 350.5 °C, while the T_5%_ of micron-HC/Eu membranes was 328.4 °C, and its T_max_ at 362.2 °C when the decomposition rate reached the fastest. This might be attributed to the fact that Eu starts to volatilize or thermally decompose at lower temperatures, resulting in an obvious early weight loss of the samples before the main decomposition stage of the membrane [40]. Nano-HC/Eu membranes and micron-HC/Eu membranes retained some solid residue, respectively, after decomposition, which was mainly attributed to the presence of calcium carbonate, the main component of HC. Furthermore, it was confirmed that HC loading was present in the fibrous membranes.

### 3.3. Water Vapor Barrier and Surface Hydrophilicity of Fibrous Membranes

**Figure 3:**
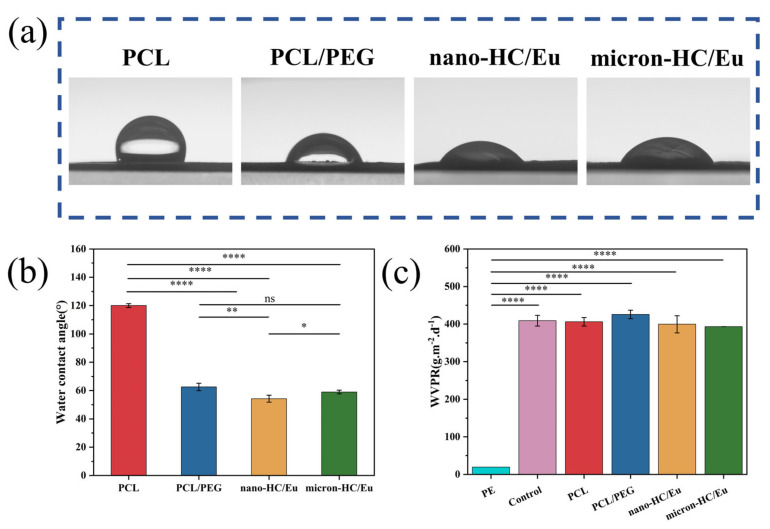
Wettability and water vapor permeability of fibrous membranes. (**a**) Representative water contact angle images of fibrous membranes; (**b**) quantitative statistics of water contact angles (*n* = 3); and (**c**) WVTR of fibrous membranes (*n* = 3). * *p* < 0.05, ** *p* < 0.01, **** *p* < 0.0001; ns, not significant.

As a wound dressing, the fibrous membranes must possess excellent hydrophilicity to achieve the rapid absorption of exudate, good breathability, and the promotion of cell growth [41]. Measuring the water contact angle facilitated the evaluation of the hydrophilicity of the membranes. Representative water contact angle images and the corresponding quantitative measurements of the membranes are shown in Figure 3a,b. The PCL fibrous membrane exhibited hydrophobicity with a water contact angle of 120.0° (>90°) [42]. The incorporation of the hydrophilic polymer PEG significantly reduced the water contact angle of PCL/PEG fiber membranes to 62.6° (<90°). This reduction is primarily attributed to PEG abundant -OH groups. The incorporation of nano-HC/Eu into the PCL/PEG membranes further decreased the water contact angle to 54.3°. These results indicate that incorporating nano-HC into the membranes enhances their hydrophilicity, thereby promoting cell adhesion and providing a more favorable physiological microenvironment for cell growth [43].

WVTR is a key factor in maintaining a moist healing environment for wound dressings. An appropriate WVTR effectively regulates the humidity of the wound microenvironment, preventing both excessive drying that impairs normal cellular physiological activity and excessive moisture that leads to exudate accumulation and subsequent infection. This creates optimal conditions for cell proliferation, migration, and tissue repair during wound healing. Figure 3c shows that the WVTR values of the micron-HC/Eu and nano-HC/Eu fibrous membranes were 392.9 g·m^−2^·d^−1^ and 399.5 g·m^−2^·d^−1^, respectively. These values were comparable to those of the blank group and were significantly higher than those of the PE group, indicating that HC incorporation did not compromise gas permeability and effectively supports vapor exchange.

### 3.4. Antibacterial and Antioxidant Functions for Infected Wound Microenvironment Regulation

In infected wounds, excessive bacterial proliferation, oxidative stress, and the dysregulated release of bioactive agents collectively impair tissue regeneration. Therefore, the ability of wound dressings to regulate the infected wound microenvironment is critical for effective healing.

**Figure 4:**
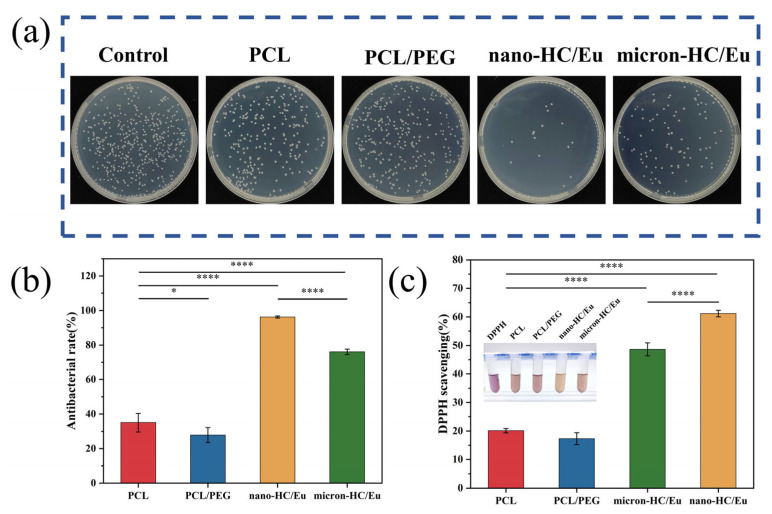
Evaluation of antibacterial and antioxidant properties of fiber membranes: (**a**) inhibitory effects of fiber membranes against *V. vulnificus*; (**b**) quantitative analysis of antimicrobial activity against *V. vulnificus* (*n* = 3); and (**c**) statistical analysis of DPPH free radical scavenging in fiber membranes (*n* = 3). * *p* < 0.05, **** *p* < 0.0001.

Bacterial infection is a major contributor to delayed wound healing [44]. Therefore, infected wounds require wound dressings with antimicrobial properties, which are critical for their effective treatment. In this study, the antimicrobial performance of fiber membranes was evaluated using a colony count method. Representative images and quantitative results of bacterial colonies after treatment with different fibrous membranes are shown in Figure 4a,b. Both micro-HC/Eu and nano-HC/Eu-loaded fiber membranes showed marked antibacterial efficacy against *V. vulnificus* compared with the PCL and PCL/PEG membranes. Among them, the antibacterial rate of the nano-HC/Eu membranes against *V. vulnificus* reached 96.2%. The enhanced antibacterial performance of the nano-HC/Eu membranes likely results from an increased specific surface area, enabling more effective interactions between the active components and bacterial cells [45]. Research indicates that eugenol effectively inhibited and killed *V. vulnificus* through multiple mechanisms, including inducing oxidative stress, disrupting cell membrane integrity, and eliminating biofilms [15]. Overall, these results demonstrated that nano-HC/Eu incorporation effectively enhanced the antibacterial performance of fibrous membranes against *V. vulnificus*, supporting their potential application as functional wound dressings for infected wound treatment.

Beyond bacterial inhibition, excessive reactive oxygen species (ROS) accumulation further exacerbates tissue damage in infected wounds. Oxidative stress is one factor that slows down the wound healing process. ROS at physiological levels participate in antibacterial defense and cell signaling. However, excessive ROS accumulation can damage surrounding tissues, and impair cell proliferation and migration [46]. Therefore, wound dressings containing antioxidants are beneficial for regulating the oxidative balance of the wound’s microenvironment. The antioxidant capacity of the fibrous membrane was quantitatively assessed by the DPPH radical scavenging assay (Figure 4c). After the incorporation of HC and Eu, the DPPH radical scavenging rate increased markedly. Among all samples, the nano-HC/Eu membranes exhibited the highest DPPH radical scavenging efficiency (61.2%). This enhancement may primarily result from phenolic hydroxyl groups in Eu, capable of donating hydrogen atoms to neutralize free radicals. In addition, the high specific surface area of nano-HC/Eu may promote more effective exposure of antioxidant components, thereby enhancing radical scavenging activity. These results indicated that the nano-HC/Eu membranes possessed favorable in vitro antioxidant capacity, which might contribute to alleviating oxidative stress in the wound microenvironment and support subsequent tissue repair during wound healing.

### 3.5. Eu Release Behavior

**Figure 5:**
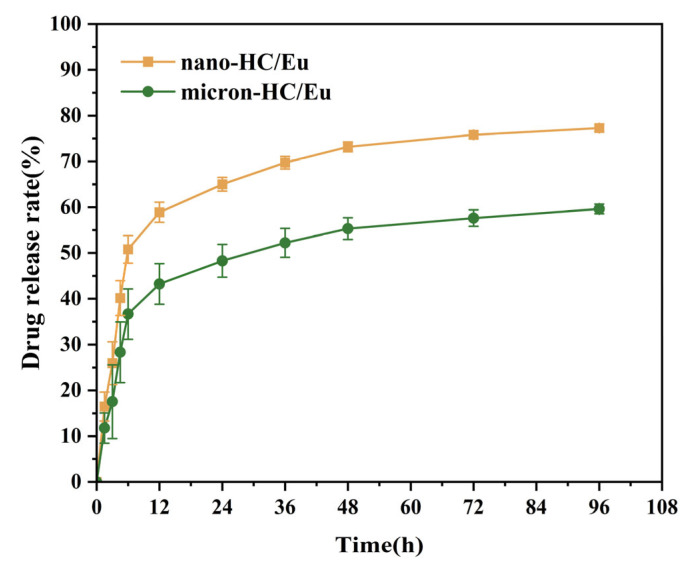
Cumulative release rate of Eu from fibrous membranes (*n* = 4).

Based on the determined maximum absorption wavelength of eugenol and its standard curve (Table S3; Figure S7), the cumulative drug release rate of the eugenol-loaded fibrous membrane at different time points was measured, and its temporal variation trend was analyzed (Figure 5). The experimental results showed that both membranes exhibited a distinct biphasic release behavior. Eu was released rapidly during the first 6 h, after which the release rate progressively decreased, transitioning to a sustained slow-release phase after 12 h. The release process approached equilibrium after approximately 96 h. It is noteworthy that the release rate of Eu from the fiber membrane containing nano-HC was significantly faster than that from the membrane containing micron-HC at the same time point comparison. This may be attributed to the smaller fibrous diameter of nano-HC/Eu compared to micron-HC/Eu, resulting in an increased surface area and thus a shorter diffusion distance leading to a faster release of Eu [47]. The biphasic release profile observed in this study, characterized by an initial burst release followed by a sustained release phase, is considered advantageous for wound dressing applications [48]. The burst release phase enables rapid antibacterial action to control initial infection, whereas the sustained release phase ensures prolonged protection throughout wound healing.

### 3.6. Cytocompatibility and Cell Migration

**Figure 6:**
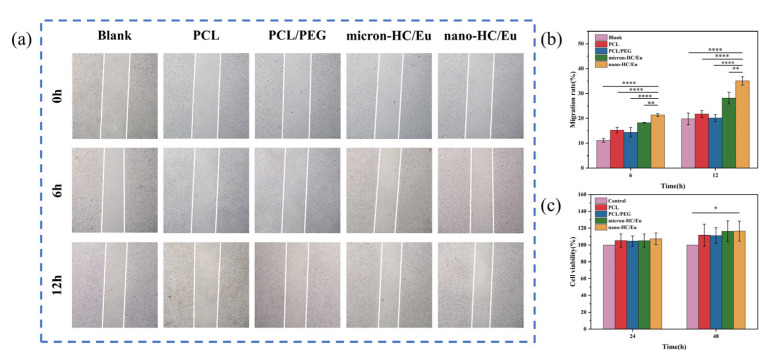
Biocompatibility of different fibrous membranes: (**a**,**b**) L929 cell scratch assay and cell mobility analysis of viability (*n* = 3); (**c**) statistical analysis of L929 cell viability (*n* = 5). * *p* < 0.05, ** *p* < 0.01, **** *p* < 0.0001.

Cell migration is a key determinant of wound healing, contributing to tissue regeneration and re-epithelialization. The impact of fibrous membranes on L929 cell migration was evaluated using a scratch assay. Cells treated with the nano-HC/Eu fibrous membranes exhibited the most pronounced migratory response (Figure 6a,b). After 12 h of incubation, the nano-HC/Eu treatment produced 35.08 ± 1.69% wound closure, a value that was significantly greater than the closure achieved by the micron-HC/Eu group. This enhanced migration suggests that the nano-HC/Eu fibrous membrane more effectively promotes cellular motility. These results demonstrated that nano-HC/Eu enhanced cell migration, a critical process in wound healing. The improved migratory performance was attributed to the smaller diameter and higher specific surface area of the nano-HC/Eu fibrous membrane, which enhanced interactions with cells and the local microenvironment, thereby promoting cell migration and facilitating subsequent tissue repair [49].

Ensuring biocompatibility is a critical requirement for antimicrobial materials intended for biomedical applications [50]. The proliferative activity of L929 cells was assessed based on the absorbance magnitude of the liquid in the well plate at 540 nm, which was proportional to the number of cells. After 24 h of co-culture with L929 cells (Figure 6c), the cell viability in the nano-HC/Eu membrane and micron-HC/Eu membrane groups exceeded that of the blank control, reaching 107.29% and 105.08%, respectively. These results indicated that neither membrane exhibited detectable cytotoxicity under the tested conditions. After 48 h of co-culture, the cell viability in both groups further increased, indicating that the fibrous membrane maintained good biocompatibility after a longer period of time. According to the cytotoxicity grading, the cytotoxicity of the fiber membrane dressing was grade 0 [51], confirming their noncytotoxic nature. These results demonstrated that the incorporation of either micron- or nano-sized HC/Eu did not adversely affect cellular viability, supporting the suitability of the prepared fibrous membranes for wound dressing applications.

### 3.7. Blood Compatibility Evaluation of Fibrous Membranes

**Figure 7:**
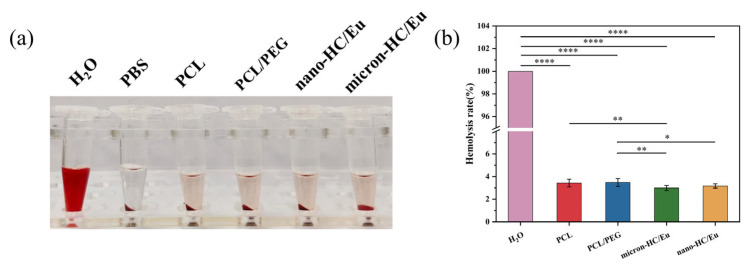
Hemocompatibility of fiber membranes. (**a**) Pictures of fiber membrane extracts and erythrocyte solutions after coincubation for 1 h; (**b**) hemolysis rate of fiber membranes (*n* = 5). * *p* < 0.05, ** *p* < 0.01, **** *p* < 0.0001.

Wound dressings come into direct contact with blood at the wound site and high rates of hemolysis (≥5%) can lead to instability and the rupture of red cell membranes, and hemolysis assays are an effective way to screen for these risks. As shown in Figure 7a, deionized water, used as the positive control, induced complete erythrocyte rupture, resulting in a bright red supernatant. In contrast, the fibrous membrane supernatant of the group containing nano-HC/Eu and micron-HC/Eu was almost colorless and transparent, with only a few ruptured erythrocytes. Quantitative hemolysis analysis further confirmed that all fiber membrane groups exhibited hemolysis rates below 5% (Figure 7b), consistent with the ISO 10993-4 international standard for the biological evaluation of medical devices [52,53]. Notably, the incorporation of either micron- or nano-sized HC/Eu did not induce a significant increase in hemolysis compared with the polymer only membranes, suggesting that the introduction of bioactive components did not compromise blood compatibility.

The above results showed that the membrane had a good blood compatibility, supporting its suitability for application as wound dressings in blood-contacting environments.

### 3.8. In Vivo Evaluation of Wound Healing in a V. vulnificus-Infected Model

**Figure 8:**
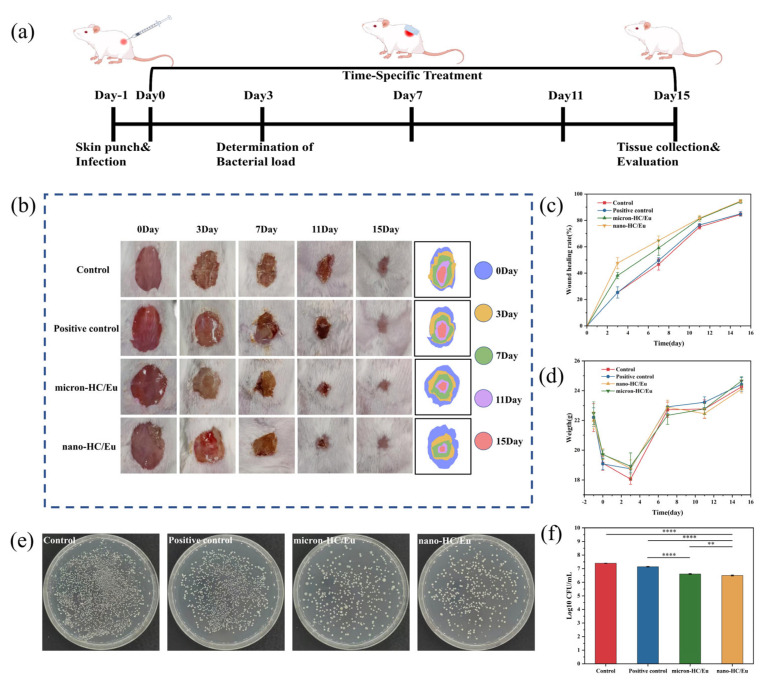
In vivo wound healing and antibacterial performance of fibrous membranes in a *V. vulnificus* infected skin wound model. (**a**) Schematic diagram of *V. vulnificus* infection modeling and treatment; (**b**) representative photographs of the wound site on days 0, 3, 7, 11, and 15 post-treatment with schematic diagrams of simulated skin wounds for each group; and (**c**) quantitative analysis of the rate of wound healing over time for each treatment group (*n* = 6). (**d**) Body weight monitoring of mice during treatment (*n* = 6); (**e**) representative photographs of bacterial colony agar plates from wound cultures on day 3 for each group (*n* = 3); and (**f**) quantification of bacterial survival based on colony forming unit (CFU) analysis (*n* = 3). ** *p* < 0.01, **** *p* < 0.0001.

To investigate the in vivo therapeutic efficacy of fibrous membranes, *V. vulnificus* infection in full thickness skin wounds was established in this study (Figure 8a). The wound healing process was recorded by taking photographs and monitored for body weight changes on days 0, 3, 7, 11, and 15, respectively (Figure 8b–d). This study initially included a total of 24 BALB/c mice. No mice died unexpectedly or required early euthanasia, and all predefined wound healing indicators and bacteriological data were successfully collected. Therefore, all data from the initially included animals were incorporated into the final analysis, with no exclusions.

At day 3, all experimental groups exhibited evident suppuration at the wound site, accompanied by a continuous decrease in body weight in the first 3 days, indicating the successful establishment of the *V. vulnificus* infection model. Both the micron-HC/Eu and nano-HC/Eu treatments produced smaller wound areas than the positive control group, suggesting that the application of the membranes might contribute to early infection control and the attenuation of acute inflammatory damage. By day 7, the wound area of each group showed a decreasing trend, indicating progression into the proliferative phase of healing. Quantitative analysis revealed a healing rate of 59.1 ± 5.6% in the micron-HC/Eu group and 64.8 ± 3.5% in the nano-HC/Eu group, respectively. The superior performance of the nano-HC/Eu membrane suggested that nanoscale HC incorporation might enhance the biological activity of the dressing, potentially through improved interfacial contact, sustained bioactive ion release, or more effective modulation of the wound’s microenvironment. At the same time, body weight recovery was observed in the fiber membrane-treated groups, indicating an overall improvement in systemic health status and further supporting the beneficial role of these dressings in infection resolution and tissue repair. By day 15, fiber membrane treatment resulted in near-complete wound closure. Healing reached 91.7 ± 4.1% in the micron-HC/Eu group and 94.7 ± 1.1% in the nano-HC/Eu group, with the nano-HC/Eu group showing more uniform epithelialization. These findings indicated that fibrous membranes loaded with nano-HC/Eu can significantly accelerate the healing of wounds infected with *V. vulnificus*.

The quantitative analysis of the *V. vulnificus* load at the wound site on day 3 (Figure 8e,f) revealed that treatment with the fibrous membranes effectively reduced the bacterial burden compared to the blank control. Specifically, wounds treated with the nano-HC/Eu membrane exhibited a significantly lower bacterial count, achieving greater bacterial clearance than the micron-HC/Eu membrane (*p* < 0.05). As previously mentioned, the nano-HC/Eu film released eugenol more rapidly and in greater quantities, enabling more effective contact with bacteria. Effectively reducing the bacterial load during the critical early stages of wound healing was essential for preventing infection spread and creating a favorable environment for tissue regeneration.

**Figure 9:**
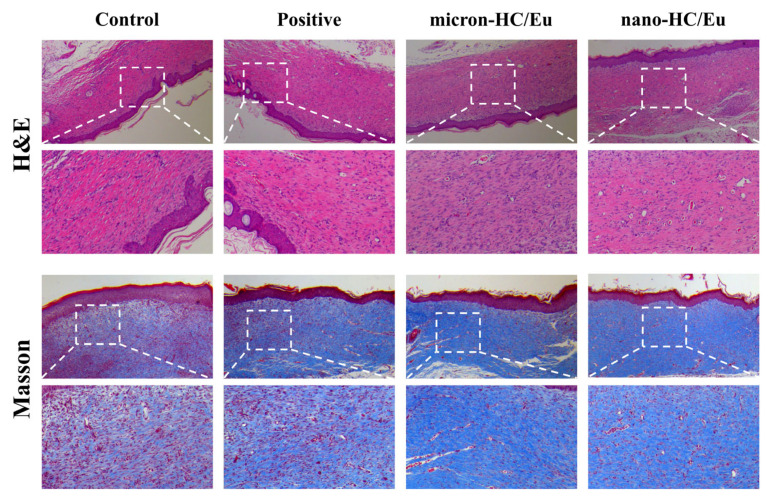
Wound healing in *V. vulnificus*-infected wounds was evaluated on day 15 using H&E and Masson’s trichrome staining.

To further investigate the healing of wounds in *V. vulnificus*-infected mice, histological examinations were performed on day 15 (Figure 9). H&E staining revealed that wounds treated with the nano-HC/Eu fibrous membrane had formed continuous, complete stratified epithelial coverage. Increased cellularity and vascularization were observed with the inflammatory response largely subsided. Masson staining further revealed abundant, orderly arranged deep blue collagen fibrous deposits within the dermis of wounds treated with the nano-HC/Eu membrane. These results indicated that treatment with the nano-HC/Eu membrane was associated with enhanced re-epithelialization and collagen deposition during wound healing [54].

Collectively, these results demonstrated that the HC/Eu-loaded fiber membranes not only effectively suppressed *V. vulnificus* proliferation during the early stage of infection but also facilitated subsequent wound repair.

## 4. Conclusions

In this study, a multifunctional fibrous membrane incorporating antibacterial and antioxidant functions was developed to modulate the wound microenvironment and promote the healing of infected wounds. The dressing effectively inhibited bacterial growth and reduced excessive ROS, thereby alleviating infection and oxidative stress-induced damage in vitro. Moreover, the sustained release of bioactive components improved cellular responses and significantly accelerated wound healing in vivo by enhancing collagen deposition and overall tissue regeneration. In summary, this study introduced a multifunctional fibrous membrane with promising potential for treating marine bacterial infection wounds and underscored the value of MTCM-derived components in regenerative medicine. Nevertheless, while the nano-HC/Eu membrane showed encouraging clinical prospects, additional long-term in vivo studies and mechanistic investigations are necessary to further validate its therapeutic efficacy and facilitate clinical translation. This study’s limitations included its potential bias (blinding/operator) and limited translatability; it requires larger independent confirmation/other models.

## Data Availability

All data needed to support the conclusions in the paper are presented in the manuscript and/or the Electronic Supplementary Materials. Additional data related to this paper may be requested from the corresponding author upon request.

## Supplementary Materials

The following supporting information can be downloaded at https://www.mdpi.com/article/10.3390/polym18060704/s1, Table S1: Table of D50 particle size variation in milled nano-HC; Figure S1: Distribution of micron- and nano-sized HC particle sizes; Figure S2: Characteristics of micron- and nano-HC. (a) SEM image of micron- and nano-HC; (b) FTIR spectroscopy of 80 mesh, micron- and nano-HC; Table S2: Single-factor experimental design parameters for preparing fibrous membranes; Figure S3: SEM micrographs and fibrous diameter distribution analysis of fibrous membranes prepared with varying PCL/PEG ratios; Figure S4: SEM micrographs and fibrous diameter distribution analysis of fibrous membranes prepared with varying HC contents at a fixed eugenol loading of 0.5%; Figure S5: SEM micrographs and fibrous diameter distributions of fibrous membranes prepared with varying eugenol concentrations at a fixed HC addition rate of 1.5%; Table S3: Eugenol standard curve data (UV-Vis spectrophotometry at 282 nm); Figure S6: (a) Viscosity of the spinning solution; (b) Electrical Conductivity of the Spinning Solution; Figure S7: Spectroscopic characterization and quantitative analysis of eugenol. (a) UV-Vis absorption spectra of eugenol in PBS and 0.2% Tween 80; (b) standard curve between absorbance measured at 282 nm and concentration of eugenol. References [36,55] are cited in the Supplementary Materials.

## Abbreviations

The following abbreviations are used in this manuscript:

HC	*Haliotidis Concha*
*V. vulnificus*	*Vibrio vulnificus*
Eu	Eugenol
PCL	Polycaprolactone
PEG	Polyethylene glycol
PBS	Phosphate-buffered saline
DCM	Dichloromethane
DMF	Dimethylformamide
SEM	Scanning electron microscopy
TEM	Transmission electron microscope
FTIR	Fourier transform infrared spectroscopy
WVIR	Water vapor transmission rate
TG	Thermogravimetric
DTG	Differential thermogravimetric
ROS	Reactive oxygen species
CCK-8	Cell Counting Kit-8
MTCM	Marine traditional Chinese medicines
