# Supplementary material for: Synergistic Antibacterial and Pro-Healing Effects of a Novel Eugenol/Nano-Haliotidis Concha Electrospun Membrane for Vibrio vulnificus-Infected Wound
Source: Polymers (Basel). 2026 Mar 13;18(6):704. doi: 10.3390/polym18060704 (PMC13030148; doi:10.3390/polym18060704)
Supplement: Supplementary file 1 [file polymers-18-00704-s001.zip › polymers-4166604-supplementary.pdf]

Synergistic Antibacterial and Pro-Healing Effects of a Novel Eugenol/Nano-*Haliotidis Concha* Electrospun Membrane for *Vibrio vulnificus*-Infected Wound

Fuyu Zhao [1,2], Xianjun Fu [1,2], Wuyi Zhou [1,2,3,*] and Xia Ren [1,2,*]

[1] Research Institute for Marine Traditional Chinese Medicine (Qingdao Academy of Chinese Medical Sciences), The SATCM's Key Unit of Discovering and Developing New Marine TCM Drugs, Key Laboratory of Marine Traditional Chinese Medicine in Shandong Universities, Shandong University of Traditional Chinese Medicine, Jinan 250355, China; 2023111578@sdutcm.edu.cn (F.Z.); fuxianjun@sdutcm.edu.cn (X.F.)

[2] Shandong University of Traditional Chinese Medicine Qingdao Academy of Chinese Medical Sciences, Qingdao 266114, China

[3] College of Materials and Chemical Engineering, Guangdong Province Biobased Green Packaging Materials Engineering Technology Research Center, South China Agricultural University, Guangzhou 510642, China

* Correspondence: zhouwuyi@scau.edu.cn (W.Z.); 1989renxia@163.com (X.R.).

Nanometer HC was prepared by ultrasonic sanding technology. After 5 h of grinding, the average particle size was measured to be approximately 122 nm (Table S1). The particle size distribution was further analyzed using a laser particle size analyzer (Figure S1). The results indicated that the particle size of nanoscale HC was concentrated around 122 nm, whereas some insufficiently refined microscale HC exhibited a particle size of approximately 459 nm.

Table S1. Table of D_{50} particle size variation of milled nano HC.

Mass-liquid ratio	Time(h)	D_{50}(μm)
1:20	0	13.725
	1	2.208
	2	1.289
	3	1.164
	4	0.588
	5	0.122
	6	0.719

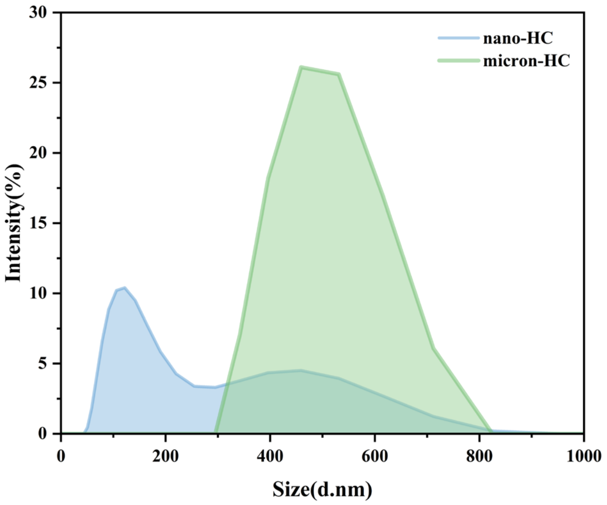

Figure S1. Distribution of micron- and nano-sized HC particle sizes.

SEM revealed that micron-sized HC particles generally exhibit irregular blocky shapes (Figure S2a), whereas nano-sized HC particles, owing to their small dimensions and high specific surface area, aggregate into clusters of fine particles under the influence of intermolecular forces. To investigate whether physical grinding induces changes in the chemical structure of HC, FTIR was employed to compare HC samples with three distinct particle sizes (Figure S2b). The results revealed that all samples exhibited C-O antisymmetric stretching vibration peaks near 1487 cm^{-1} and CO_3^{2-} out-of-plane bending vibration peaks near 857 cm^{-1}. Furthermore, the overall peak shapes and positions across all spectra demonstrated high consistency. This indicates that nanoscale processing achieves only physical size reduction without altering the fundamental molecular structure or functional groups.

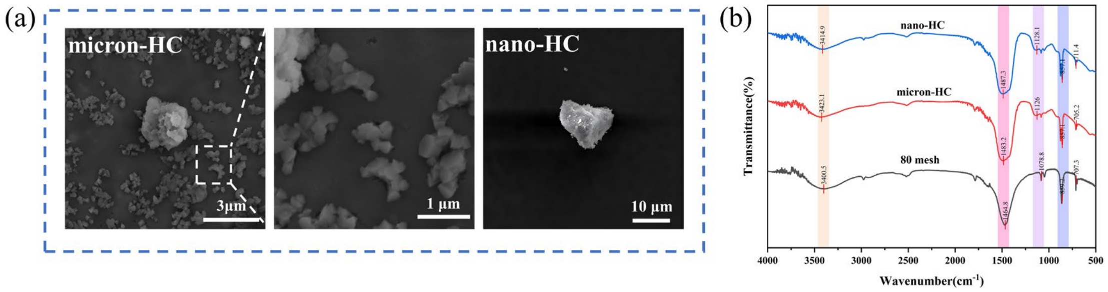

Figure S2. Characteristics of micron- and nano-HC. (a) SEM image of micron- and nano-HC; (b) FTIR spectroscopy of 80 mesh, micron- and nano- HC.

Through single-factor experiments, the process parameters for preparing the fibrous membrane were optimized and screened (Table S2), determining the optimal conditions as follows: a PCL to PEG mass ratio of 8:2, HC content of 1%, and Eu content of 0.75%. Under these conditions, the resulting fibrous membranes exhibited excellent morphological structure with uniform fibrous diameter distribution, free from curling or adhesion. Specific morphological features are illustrated in the accompanying figures (Figure S3 - S5).

Table S2. Single-factor experimental design parameters for preparing fibrous membranes.

PCL: PEG	HC addition amount(w/v)	Eugenol addition amount(v/v)	Voltage(kV)	Flow velocity(mL/h)
5:5	0.5%	0.25%	20	2
6:4	1%	0.5%	20	2
7:3	1.5%	0.75%	20	2
8:2	2%	1%	20	2
9:1	2.5%	1.25%	20	2

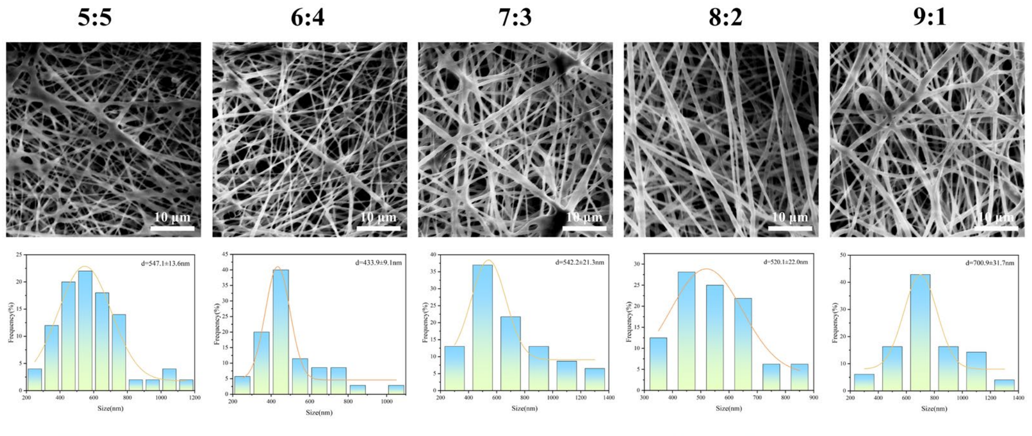

Figure S3. SEM micrographs and fibrous diameter distribution analysis of fibrous membranes prepared with varying PCL/PEG ratios.

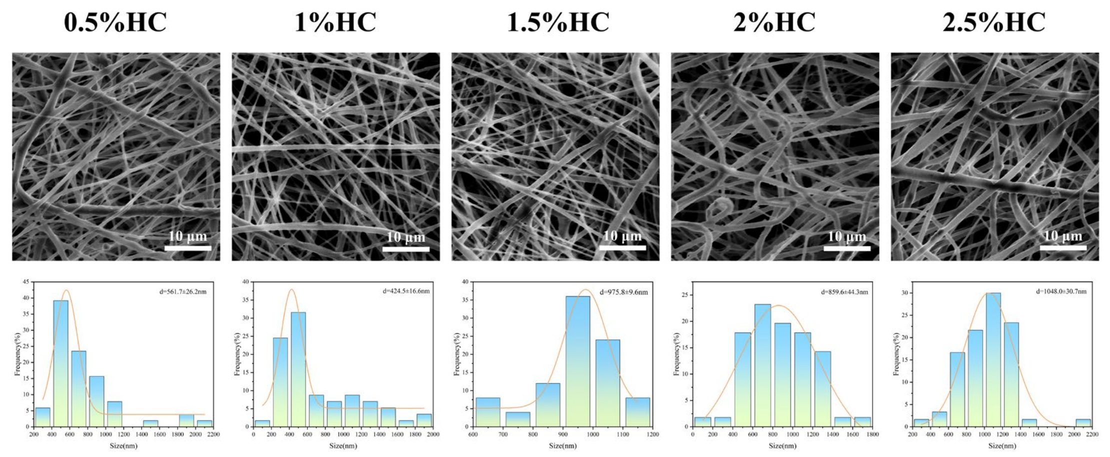

Figure S4. SEM micrographs and fibrous diameter distribution analysis of fibrous membranes prepared with varying HC contents at a fixed eugenol loading of 0.5%.

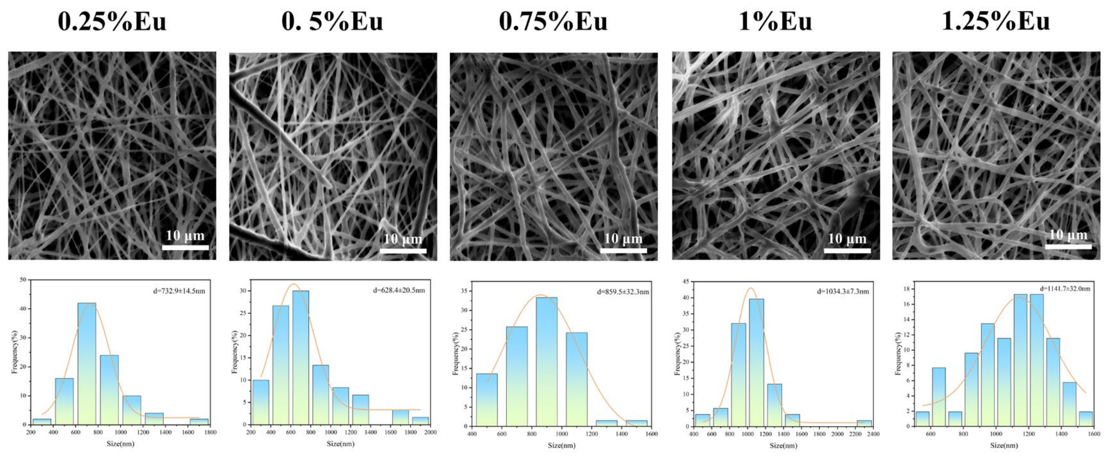

Figure S5. SEM micrographs and fibrous diameter distributions of fibrous membranes prepared with varying eugenol concentrations at a fixed HC addition rate of 1.5%.

In electrospinning, a high-voltage electric field induces charges on the surface of the spinning droplet. When the electric field force overcomes the droplet's surface tension, the droplet deforms into a Taylor cone. If the droplet's cohesive force is too weak, which is primarily determined by viscosity, the droplet directly ejects small droplets rather than producing a continuous jet. Viscosity provides the necessary viscoelasticity. Only when viscosity is sufficiently high can molecular chains become adequately entangled, allowing the jet to maintain continuity under the stretching force of the electric field without breaking into droplets. The viscosity results for each spinning solution were shown in Figure S6a. The PCL spinning solution exhibited the highest viscosity at 510 cP. This elevated viscosity imposed significant viscous resistance on the jet during electrospinning, hindering the electric field's ability to sufficiently stretch. Consequently, the jet solidified relatively quickly, resulting in thicker fibers. After adding PEG, HC and Eu, the spinning solution viscosity gradually decreased. Among them, the spinning solution viscosity for micron-HC/Eu fiber membranes was 405 cP, while that for nano-HC/Eu fiber membranes was the lowest at 402.5 cP. The decrease in viscosity from 510 cP for pure PCL to 450 cP for PCL/PEG was attributed to the

plasticizing effect of PEG, where low molecular weight PEG molecules intercalated between PCL chains, reducing chain entanglement and increasing chain mobility[1]. The further decrease in viscosity after adding HC was attributed to the effective dilution of the polymer solution by solid HC particles, thereby reducing the frequency of entanglement per unit volume. This phenomenon of viscosity reduction following the addition of solids and molecules has been reported in similar systems[2]. As the viscosity of the spinning solution decreased, its electrical conductivity increased (as shown in Figure S6b). The synergistic effect of these two factors significantly promoted fiber draw-out and refinement.

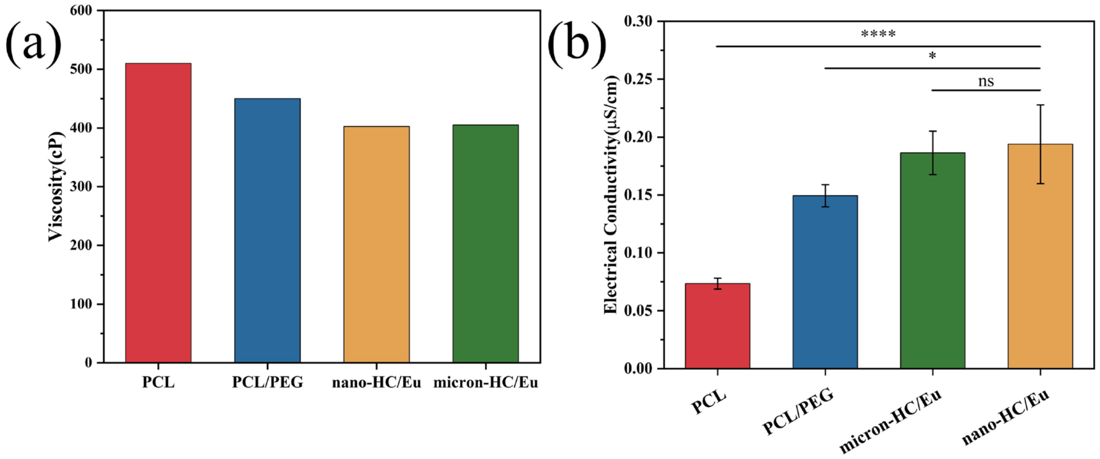

Figure S6. (a) Viscosity of the spinning solution; (b) Electrical Conductivity of the Spinning Solution.

The results showed that a full-wavelength UV-Vis scan of eugenol in PBS containing 0.2% Tween 80 revealed a maximum absorption peak at 282 nm (Figure S7a). Consequently, 282 nm was determined as the characteristic detection wavelength for subsequent quantitative analysis. Subsequently, absorbance measurements were performed at this wavelength for eugenol solutions at different gradient concentrations (Table S3). The absorbance increased progressively with increasing concentration, demonstrating good concentration dependence. Finally, a standard curve was plotted with the linear equation $y = 0.021x + 0.66$ and a linear fit coefficient of determination $R^2 = 0.995$ (Figure S7b). This indicates that the method exhibits high linearity and reliability within the defined concentration range, making it suitable for quantitative calculation and comparison of eugenol content in subsequent samples.

Table S3. Eugenol standard curve data (UV-Vis spectrophotometry at 282 nm).

Concentration(μg/mL)	Absorbance(a.u.)
4	0.745
2	0.7
1	0.685
0.5	0.669
0.25	0.667
0.125	0.663
0.0625	0.66

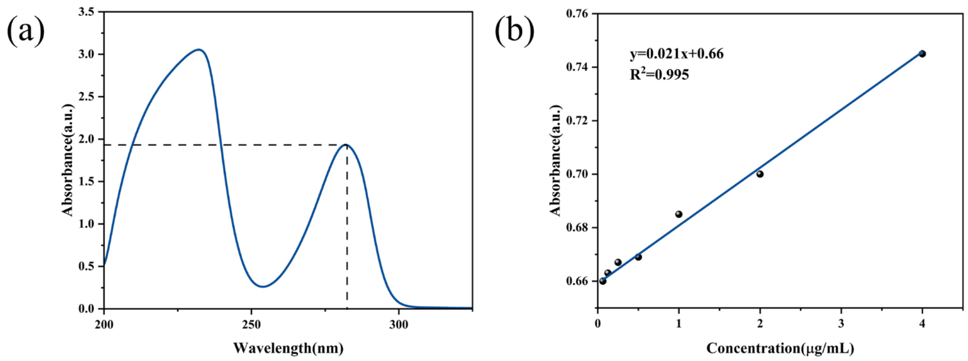

Figure S7. Spectroscopic characterization and quantitative analysis of eugenol. (a) UV-Vis absorption spectra of eugenol in PBS and 0.2% Tween 80; (b) Standard curve between absorbance measured at 282 nm and concentration of eugenol.

To minimize potential bias, the order of animal procedures and cage locations were randomized. The primary outcome measures were assessed by researchers blinded to the group allocation. During the experiment, humane endpoints were predefined. Mice exhibiting severe lethargy with unresponsiveness to stimuli or self-mutilation were excluded from the study. Any mouse exhibiting predetermined humane endpoints would be immediately euthanized and excluded from subsequent analyses. These endpoints included signs of systemic sepsis due to wound infection, weight loss exceeding 20% of initial body weight, or wound necrosis spreading beyond the predefined study scope. These criteria were established prior to the experiment's commencement.

References

1. Pan, L.;Yang, J.H.;Xu, L.Preparation and Characterization of Simvastatin-Loaded PCL/PEG Nanofiber Membranes for Drug Sustained Release. *Molecules* **2022**, *27*, 7158.
2. Scaffaro, R.;Settanni, L.;Gulino, E.F.Release Profiles of Carvacrol or Chlorhexidine of PLA/Graphene Nanoplatelets Membranes Prepared Using Electrospinning and Solution Blow Spinning: A Comparative Study. *Molecules* **2023**, *28*, 1967.
